# A method for quantitation of apoplast hydration in Arabidopsis leaves reveals water-soaking activity of effectors of *Pseudomonas syringae* during biotrophy

**DOI:** 10.1038/s41598-022-22472-x

**Published:** 2022-11-01

**Authors:** Gayani Ekanayake, Reid Gohmann, David Mackey

**Affiliations:** 1 Department of Horticulture and Crop Science, The Ohio State University, Columbus, OH 43210 USA; 2 Molecular, Cellular, and Developmental Biology Program, Ohio State University, Columbus, OH 43210 USA; 3 Department of Molecular Genetics, The Ohio State University, Columbus, OH 43210 USA; 4 Center for Applied Plant Sciences, The Ohio State University, Columbus, OH 43210 USA

**Keywords:** Biotic, Effectors in plant pathology

## Abstract

The plant apoplast has a crucial role in photosynthesis and respiration due to its vital function in gas exchange and transpiration. The apoplast is also a dynamic environment that participates in many ion and nutrient transport processes via plasma membrane-localized proteins. Furthermore, diverse microbes colonize the plant apoplast, including the hemibiotrophic bacterial pathogen, *Pseudomonas syringae* pv. tomato (*Pto*) strain DC3000. *Pto* DC3000 initiates pathogenesis upon moving through stomata into the apoplast and then proliferating to high levels. Here we developed a centrifugation-based method to isolate and quantify the apoplast fluid in Arabidopsis leaves, without significantly damaging the tissue. We applied the simple apoplast extraction method to demonstrate that the *Pto* DC3000 type III bacterial effectors AvrE1 and HopM1 induce hydration of the Arabidopsis apoplast in advance of macroscopic water-soaking, disruption of host cell integrity, and disease progression. Finally, we demonstrate the utility of the apoplast extraction method for isolation of bacteria proliferating in the apoplast.

## Introduction

A common symptom of pathogenic infections is water-soaking (WS), a condition in which the apoplast becomes sufficiently hydrated to appear macroscopically water-logged. Pathogens have diverse lifestyles, ranging from biotrophs that complete their lifecycle by feeding on living plant cells to necrotrophs that feed on dead plant cells. Hemibiotrophic bacterial pathogens, such as *Pseudomonas syringae* pv. tomato (*Pto*) strain DC3000 that parasitizes tomato or Arabidopsis, proliferate among initially viable and later dying and dead plant cells^1^. The timing of apoplast hydration relative to macroscopic WS and the onset of necrosis during plant disease represents a major knowledge gap in plant pathology.

The plant apoplast is the matrix in which endophytes reside and many pathogens, such as *Pto* DC3000, proliferate and cause disease. The availability of water in the apoplast is key to the constituency and abundance of microbes. Water-limiting conditions are a feature of effective defense responses against pathogenic microbes^2^. Pathogens deploy virulence factors to promote water availability^3,4^. For example, *Pto* DC3000 deploys type III-secreted effectors (T3Es) encoded within the conserved effector locus (CEL), AvrE1 and HopM1, that induce WS and are essential for full disease progression^1,5^. Highlighting the importance of WS during plant disease, CEL-encoded AvrE-family T3Es are present in and key to virulence of diverse genera of phytopathogenic bacteria, including *Pseudomonas*, *Pantoea*, *Erwinia*, *Xanthomonas*, *Ralstonia*, and *Pectobacteria*^6–8^. Furthermore, transcription activator-like (TAL) effectors found in *Xanthomonas* cause both increased nutrient availability and WS in the apoplast of host plants^7^. Interestingly, the induction of WS, and perhaps associated increases in nutrient availability, is sufficient to cause outgrowth of normally non-pathogenic endophytes^8^. Conversely, beneficial endophytes can alter the apoplast to render plants more resistant to biotic or abiotic stresses^9^. Thus, methods to understand the composition of the apoplast, including its extent of hydration and composition, will enable an advanced understanding of plant–microbe interactions.

Numerous centrifugation-based approaches have been used to extract apoplast fluid from plant leaves^10–14^. However, the plant species, the age of the tissue, and the ultimate goal of the experiments leads to substantial differences in the methods. Here we establish a simple method for apoplast fluid extraction from the leaves of vegetatively growing Arabidopsis plants. The procedure enables quantitation of apoplast hydration, which is the extent to which the apoplast is filled with fluid, in advance of macroscopic WS. Using this method, we demonstrate that, during *Pto* DC3000 infection, AvrE1 and HopM1 promote apoplast hydration at a time that correlates with bacterial proliferation and precedes necrosis. We additionally demonstrate the utility of the method for isolation of bacteria from within the apoplast and for analysis of the apoplast contents.

## Results

### Apoplast extraction from Arabidopsis leaves

**Figure 1:**
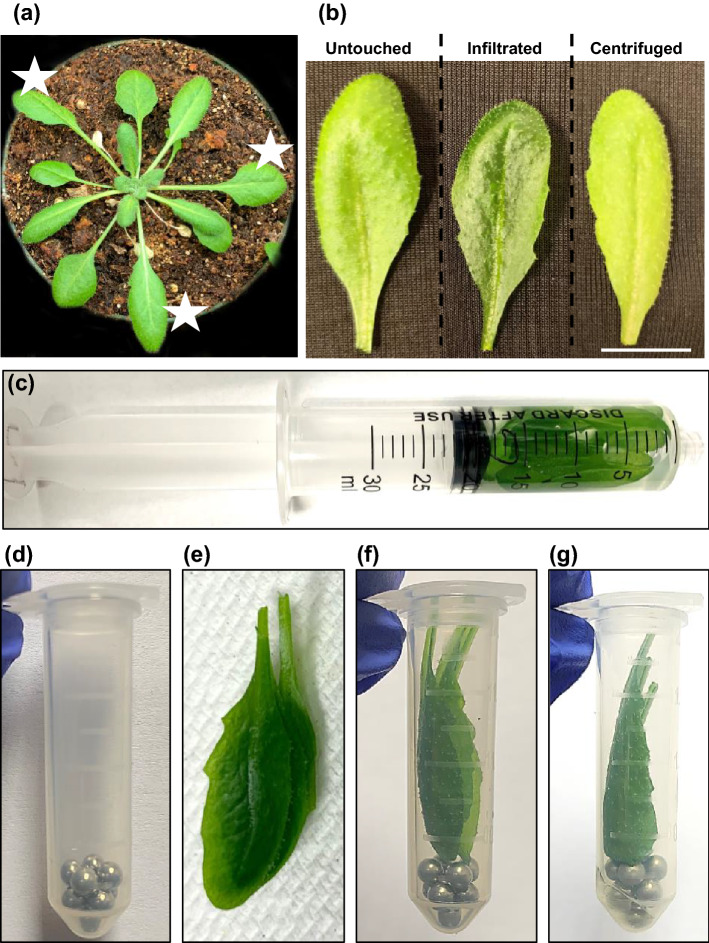
Procedure for Arabidopsis apoplast extraction. (**a**) Three leaves from 5 week-old Arabidopsis plants at a similar developmental stage (stars) were used for the assay. (**b**) Representative images of untouched, vacuum-infiltrated and centrifuged leaves (Bar = 1 cm). (**c**) Nine leaves were harvested from three plants and vacuum-infiltrated with apoplast wash fluid in a 30 cc syringe. (**d**) Two mL tube with nine 3.2 mm metal beads. Three vacuum-infiltrated leaves were blotted dry and nested together (**e**) and inserted into a 2 mL tube with metal beads (**f**). (**g**) Two mL tube with leaves after the centrifugation.

An extraction method was developed to isolate apoplast fluid from leaves of ~ 5 week-old Arabidopsis plants. Three fully expanded leaves per plant, typically leaves 8, 9, and 10 (Fig. 1a), were harvested, along with about 2 mm of the petiole, using scissors (Fig. 1b). Immediately after collection, the leaves were weighed to record the Initial Weight (IW). For each sample, nine leaves from three different plants were weighed together to minimize error. The leaves were inserted into a 30 cc syringe filled with ~ 20 mL of apoplast extraction solution (Milli-Q water) (Fig. 1c) and vacuum-infiltrated by ejecting the air and then blocking the inlet and firmly pulling on the syringe barrel 5 times for ~ 5 s per pull. Thorough infiltration of the leaves was apparent based on their uniformly darker color, translucent appearance, and lack of buoyancy compared to before infiltration (Fig. 1b). The surfaces of these infiltrated leaves were then dried by blotting with tissue paper wipes and immediately weighed to record the After Infiltration Weight (AIW). Prior to harvesting the leaves, sets of three 2 mL tubes for each nine-leaf sample were prepared with nine sterile 3.2 mm metal beads at the bottom of each tube (Fig. 1d). After recording the AIW, the leaves were split into three sets of three leaves, stacked together (Fig. 1e), and inserted into the tubes prepared for the centrifugation (Fig. 1f). Nesting of the leaves and their support atop the metal beads helped prevent damage during the centrifugation and the beads also permitted complete separation of the extracted apoplast fluid from the leaves (Fig. 1g). After processing each nine-leaf sample from harvest to IW determination, infiltration, AIW determination, and insertion into three processing tubes with beads, these tubes were kept on ice until all additional samples were processed. Then, all the tubes were centrifuged simultaneously at designated relative centrifugal forces (rcf) at 4 °C. Following the spin, the groups of nine leaves were weighed immediately upon removal from their tubes to determine the After Spin Weight (ASW).

**Figure 2:**
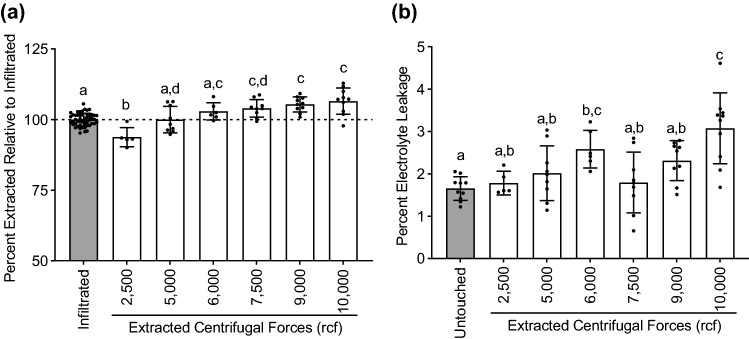
Evaluation of centrifugal force on Arabidopsis leaf apoplast extraction. (**a**, **b**) 5 week-old Arabidopsis leaves were vacuum-infiltrated with Milli-Q water and spun at different centrifugal forces for 10 min at 4 °C. (**a**) The percent of apoplast fluid extracted by centrifugation at the indicated rcfs, relative to the infiltrated amount, was calculated. (**b**) Percent electrolyte leakage was measured after centrifugation at the indicated rcfs. For each experiment, n = 2 or 3 samples/centrifugation speed, with each n consisting of 9 different leaves collected from 3 different plants. The experiment was performed 3 times and combined data (n = 6 to 9) is shown. Error bars are standard deviations and different letters denote statistically significant difference based on ordinary one-way ANOVA with Tukey’s multiple comparisons test (*p* < 0.05).

The impact of centrifugation force on yield and cellular integrity was assessed (Fig. 2). The goal was to identify the centrifugation force that maximized yield of extracted apoplast with minimal or no damage to the cellular integrity of the leaf tissue. The apoplast from leaves processed as described above was collected by centrifugation for 10 min at various rcfs ranging from 2500 to 10,000. The efficiency of extraction was determined by comparing the total amount of fluid removed from the leaves during the spin to the amount of apoplast extraction solution initially introduced into the leaves (Fig. 2a). We found that the most accurate way to determine the yield from the spin was to calculate the difference in leaf weight before and after the spin (AIW-ASW). Attempts to measure the amount of fluid in the bottom of the tubes gave lower values, likely due to liquid adherence to the large surface area of beads and tubes combined with evaporation from those surfaces. Similarly, the amount of apoplast extraction solution introduced into the leaf during the procedure was calculated as the difference in weight before and after the infiltration (AIW-IW). The amount of infiltrated apoplast extraction solution (per leaf IW) was set to 100% and the yields at the different rcfs were calculated. The extracted liquid exceeded 100% at 6000 rcf and plateaued at higher rcfs, indicating full recovery of both the infiltrated apoplast extraction solution and fluid existing in the apoplast at the time of leaf harvest (Fig. 2a).

To determine the effect of spinning at the different rcfs on cellular integrity within the leaf tissues, we measured electrolyte leakage from the leaves after the spins (Fig. 2b). The nine leaves were floated in 25 mL of Milli-Q water for 1 hour at room temperature and then conductivity of the bath solution was measured. Conductivity was measured again after the tubes containing the leaves were placed in a boiling water bath for 20 min to establish the maximum electrolyte leakage, which was set to 100%. Electrolyte leakage did not differ among any of the centrifugation speeds up to 9000 rcf. However, at 10,000 rcf, electrolyte leakage did increase significantly, which likely correlated with the observed crushing of the leaves into the metal beads. Additionally, when using larger leaves as is desirable for experiments with bacterial or other infiltration prior to the apoplast isolation, leaf tips were often observed to tear off at rcfs above 7500. Therefore, we selected 7500 rcf for 10 min at 4 °C as the optimal centrifugation condition for quantitative yield without damage to the leaf tissue and corresponding contamination of the apoplast extracts. This condition was used for all subsequent experiments.

### Application of apoplast extraction method to quantitate apoplast hydration during biotrophic growth of *Pto* DC3000

**Figure 3:**
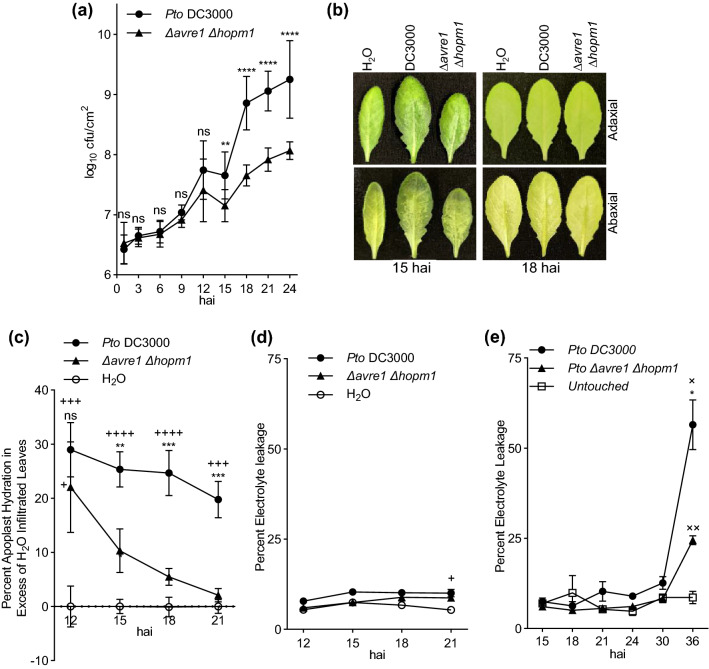
Timing of AvrE1- and HopM1-dependent bacterial growth, water-soaking, apoplast hydration, and loss of plant cell integrity. (**a**–**e**) Leaves of 5 week-old Col-0 plants were syringe-infiltrated with *Pto* DC3000 or *∆avre1 ∆hopm1* (at OD_600_ = 0.2) or with sterile Milli-Q water. (**a**) Bacterial growth over time was assessed by serial dilution plating as log_10_ colony-forming units (cfu) per leaf area. For each experiment, n = 6 samples/bacteria/time point, with each n consisting of 2 leaf discs taken from 2 different leaves. The experiment was performed 4 times and combined data (n = 24) is shown. (**b**) Representative images of the abaxial and adaxial surfaces of leaves at 15 and 18 hai. (**c**) Apoplast hydration was measured at the indicated time points and is presented as percent apoplast hydration in excess of water treated leaves. For each experiment, n = 3 samples/treatment/time point, with each n consisting of 9 leaves taken from 3 different plants. The experiment was performed 5 times and combined data (n = 15) is shown. (**d**) Percent electrolyte leakage from samples of Fig. 3c. For each replicate, n = 3 samples/treatment/time point, with each n consisting of 2 punches taken from 2 different leaves. Shown is combined data from four of the five replicates (n = 12). (**e**) Percent electrolyte leakage over a longer time course than in Fig. 3c. For each experiment, n = 3 samples/treatment/time point, with each n consisting of 2 punches taken from 2 different plants. Combined data from two experiments (n = 6) are shown. Values are means ± SEM and statistically significant differences are based on Welch’s t-test. For comparisons between *Pto* DC3000 and *∆avre1 ∆hopm1*; **** *p* < 0.0001; *** *p* < 0.0009; ** *p* < 0.005; * *p* < 0.05. For comparisons of bacterial strains and H_2_O; + + + + *p* < 0.0001; + + + *p* < 0.0009; + + *p* < 0.005; + *p* < 0.05. For comparisons of bacterial strains and untouched; × × *p* < 0.005; × *p* < 0.05.

Natural bacterial infections are not spatially or temporally uniform. Thus, to assess apoplast hydration synchronously at the scale of whole leaves, we hand-infiltrated leaves with a high titer (1 × 10^8^ cfuU/mL) of bacteria. We sought to determine the timing of apoplast hydration, WS, and necrosis induced by the AvrE1 and HopM1 T3Es relative to their support of bacterial proliferation. Thus, we first determined the timing of growth of *Pto* DC3000 and the ∆*avre1 ∆hopm1* double mutant strain (Fig. 3a) following high titer infiltration. During the first 12 hours after infiltration (hai), the two bacterial strains each increased in number by approximately 10-fold and did not significantly differ in abundance. The number of *Pto* DC3000 cells increased by > 100-fold by 18 hai and continued to proliferate through 24 hai. The number of ∆*avre1 ∆hopm1* cells increased through 24 hai, but was significantly reduced relative to *Pto* DC3000 at and beyond 15 hai.

In addition to promoting growth of *Pto* DC3000, AvrE1 and HopM1 also induce macroscopic WS and necrosis in infected host tissues. The concept of apoplast hydration is based on the extent of liquid in the apoplast that is physically separate from the cytosol of the plant cells, thus the loss of cell integrity during necrosis confounds measurement of apoplast hydration. Thus, we sought to establish the timing of WS and apoplast hydration relative to necrosis. WS symptoms apparent at 15 hai with a high titer of *Pto* DC3000 were more prominent in the abaxial than adaxial surface of the leaf (Fig. 3b). WS was much less prominent in leaves at 15 hai with buffer or ∆*avre1 ∆hopm1* and was no longer apparent at 18 hai for any of the three treatments (Fig. 3b).

We next used the apoplast extraction procedure described above (in Figs. 1 and 2) to determine the effect of AvrE1 and HopM1 on apoplast hydration from 12 to 21 hai with water or a high titer of *Pto* DC3000 or ∆*avre1 ∆hopm1*. The percent apoplast hydration was calculated by dividing the amount of liquid in the apoplast of the sampled leaves by the total capacity of the apoplast of those leaves:

$$Percent\; apoplast \;hydration = \frac{IW - ASW}{{AIW - ASW}}*100$$

Water-soaking is apparent following the initial hand-infiltrations (at t = 0) and then apoplast hydration decreases as the excess fluid in the apoplast evaporates (Supplemental Fig. 1). To reveal the influence of bacterial infection and of AvrE1 and HopM1 on apoplast hydration, we determined the percent apoplast hydration following high titer infiltration of *Pto* DC3000 or ∆*avre1 ∆hopm1* in excess of that following water-infiltration (Fig. 3c). Both bacterial strains caused apoplast hydration that was elevated relative to water-infiltration at 12 hai, likely contributing to the growth of each strain during this period (Fig. 1a). Consistent with the macroscopic observations (Fig. 3b), the rapid “drying down” of the apoplast between 12 and 21 hai likely resulted from stomatal opening after the lights turned on at ~ 14 hai (Supplemental Fig. 1)^15,16^. The contribution of AvrE1 and HopM1 to apoplast hydration became apparent after the lights came on; *Pto* DC3000 maintained a higher level of apoplast hydration than ∆*avre1 ∆hopm1* by 15 hai that persisted until at least 21 hai. To assist in utilization of the apoplast hydration assay, raw data for one replicate of this experiment is provided (Supplemental Table 1). Ion leakage measurements on the samples from Fig. 3c demonstrated that tissue integrity was maintained during the experiment, as expected (Fig. 3d). Apoplast hydration measurements were not made later than 21 hai because the bacterial infections had begun to compromise the structural integrity of the leaves such that they could not withstand the rcf of the apoplast extraction procedure. A longer time course revealed increases in electrolyte leakage from bacteria infiltrated samples later than 30 hai, corroborating macroscopic observations of necrosis (Fig. 3e).

Our calculations of apoplast hydration are based on measuring the weight of the apoplast fluid, including the bacterial biomass. To determine the significance of the bacterial biomass to these calculations, we weighed the pellets of *Pto* DC3000 and ∆*avre1* ∆*hopm1* as well as the bacteria-free apoplast fluids at 15 hai. The data revealed that the bacteria compose ~ 1% by mass of the isolated apoplast fluid (Supplemental Fig. 2). Given that the measurements of apoplast hydration at 15 hai with each bacteria have standard errors of at least 10%, we concluded that the bacteria did not significantly affect these values. Furthermore, given that the bacteria consist predominately of water derived from within the plant tissue, it could be considered appropriate to include the bacteria in the calculation of apoplast hydration.

The data in Fig. 3 collectively indicate that AvrE1 and HopM1 promote apoplast hydration, WS, and bacterial proliferation prior to their induction of necrosis. Notably, the timing of AvrE- and HopM1-induced apoplast hydration corresponds to the increased growth of *Pto* DC3000, relative to ∆*avre1 ∆hopm1*. Thus, effector-induced apoplast hydration and WS do not result from “leakage” of cytosol contents from necrotic plant cells, but rather are features of the early, biotrophic phase of *Pto* DC3000 infection of Arabidopsis leaves.

### Use of the apoplast isolation procedure to harvest bacteria from within Arabidopsis leaves

**Figure 4:**
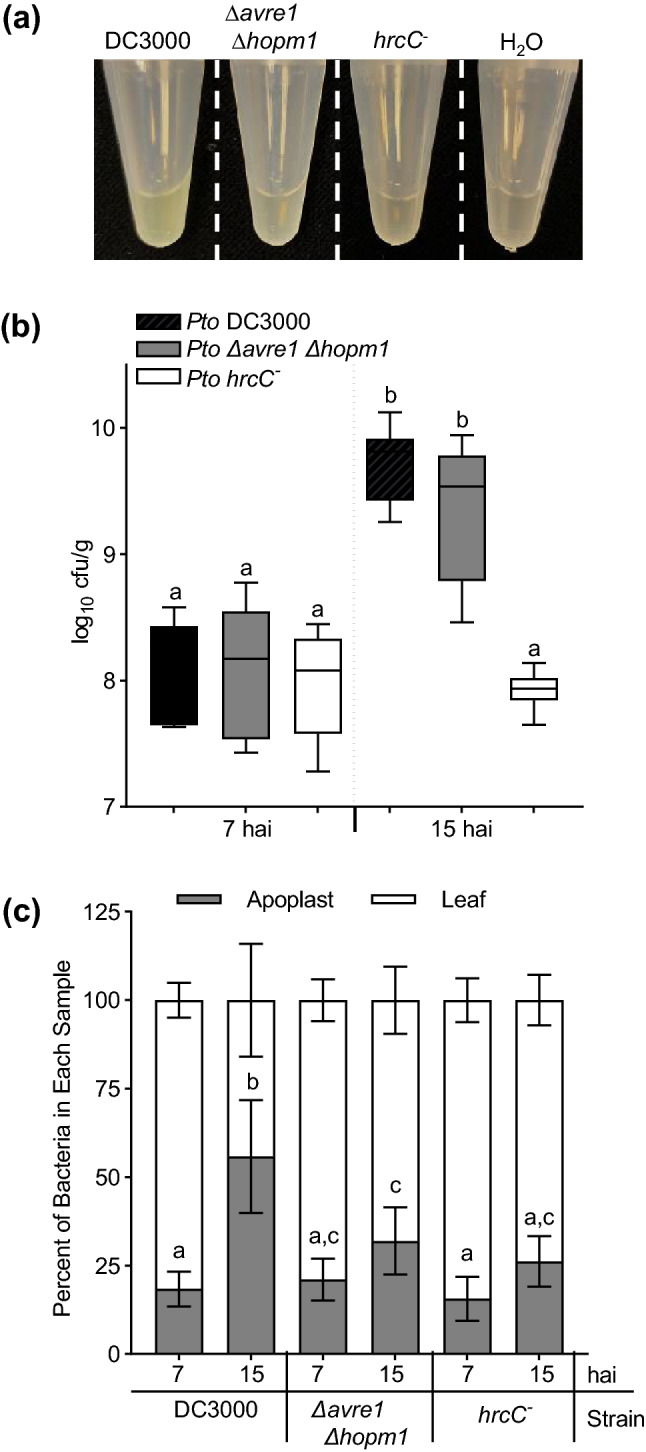
Determination of the efficiency of recovery of leaf-resident bacterial cells in apoplast fractions. (**a**–**c**) Three leaves of 5 week-old Arabidopsis plants were syringe-infiltrated with *Pto* DC3000, *∆avre1 ∆hopm1* or *Pto hrcC*^*−*^ (at OD_600_ = 0.2). (**a**) Apoplast fluid with bacteria extracted at 15 hai; note differing turbidity of samples. (**b**) Bacterial growth per tissue mass at 7 hai and 15 hai from combined measurement of bacteria in apoplast extracts and remaining in leaves. For each experiment, n = 3 samples/bacteria/time point, with each n consisting of 3 leaf discs taken from 3 different plants. The experiment was performed 3 times and combined data (n = 9) is shown. Boxes indicate the median and interquartile range and whiskers indicate minimum and maximum data distribution. (**c**) Distribution of bacteria between the apoplast and remaining leaf samples at 7 hai and 15 hai. For each experiment, n = 3 samples/fraction/bacteria/time point, with each n consisting of 3 different leaves from 3 different plants. The experiment was performed 3 times and combined data (n = 9) is shown. Error bars are standard deviations; different letters denote statistically significant difference between apoplast fractions based on ordinary one-way ANOVA with Tukey’s multiple comparisons test (*p* < 0.05).

As already discussed, the apoplast isolation procedure also collects leaf-resident bacteria (Fig. 4a). The ability to isolate bacteria directly from the plant apoplast will enable studies of their constituency, physiology, transcriptome, proteome, etc., immediately following their residency within the host tissue. Consistent with the higher level of growth of *Pto* DC3000 relative to ∆*avre1 ∆hopm1*, the turbidity of the apoplast fluid isolated from *Pto* DC3000-infected leaves was greater than that isolated from ∆*avre1 ∆hopm1*-infected leaves. Rather than simply considering qualitatively the turbidity of the bacterial extractions, we sought to measure the efficiency of isolation of *Pto* DC3000 and ∆*avre1 ∆hopm1*, as well as a *hrcC-* mutant of *Pto* DC3000 that is unable to deliver any T3Es. First, we measured the growth of these three strains following their high titer infiltration into Arabidopsis leaves (Fig. 4b). In this experiment, the total number of bacteria per mass of tissue was determined by combining the numbers present in the isolated apoplast fluid and remaining in the leaves following apoplast fluid isolation. In total, all three strains were present at comparable numbers per gram of tissue at 7 hai. By 15 hai, *PtohrcC-* had not proliferated significantly beyond its levels at 7 hai. This contrasts with *Pto* DC3000 and ∆*avre1 ∆hopm1*, which continued to proliferate between 7 and 15 hai.

Next, we analyzed the data to assess the percent of bacteria isolated with the apoplast fluid (Fig. 4c). At 7 hai, the percent of bacteria isolated in the apoplast fluid was 15 to 21% and did not differ significantly between the three strains. By 15 hai, the percent of bacteria isolated in the apoplast increased in correspondence with the extent to which they have proliferated. More than 55% of *Pto* DC3000, which had proliferated to the highest level and would be anticipated to further outgrow ∆*avre1 ∆hopm1* (Fig. 3a), was isolated in the apoplast at 15 hai. For ∆*avre1 ∆hopm1*, which is proliferating less rapidly than *Pto* DC3000, and *Pto hrcC-*, which is not proliferating, the percent of cells isolated with the apoplast fluid remained low and did not differ significantly from at 7 hai. Thus, the actively proliferating bacteria were isolated more efficiently than were recently introduced or slowly or non-proliferating bacteria.

### Evaluation of the apoplast extraction efficiency and cellular integrity with different apoplast wash solutions

**Figure 5:**
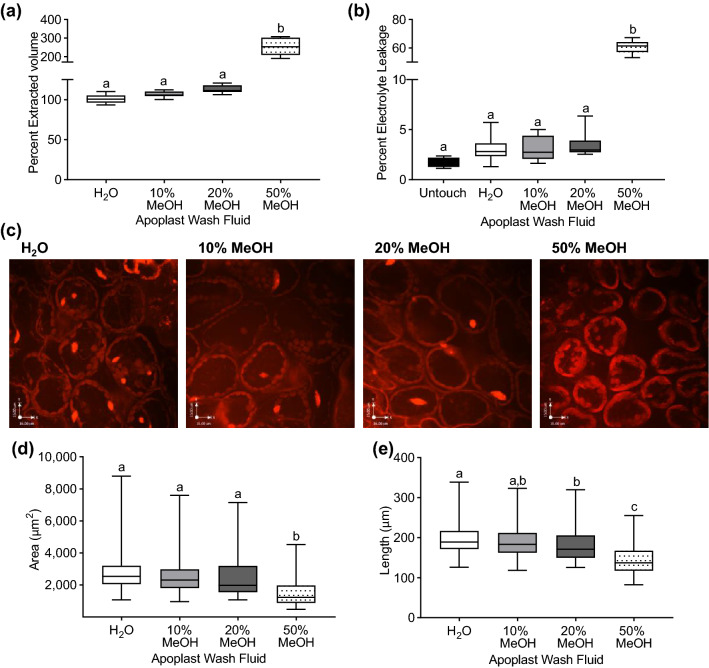
Evaluation of apoplast wash fluids. (**a**) Percent extracted volume of apoplast from Arabidopsis leaves after vacuum-infiltration with H_2_O, 10%, 20% or 50% methanol. For each experiment, n = 3 samples/infiltrate and combined data from three independent experiments (n = 9) is shown. (**b**) Percent electrolyte leakage from untouched leaves and from the post-processing leaves of (**a**). (**c**) Fully expanded, 5.5 week-old Arabidopsis leaves were vacuum-infiltrated with H_2_O, 10%, 20% or 50% methanol and leaf punches were stained with 10 µg/µl propidium iodide (PI) for 30 min at 26 °C. The leaf punches were imaged with a spinning disk confocal microscope. Shown are representative maximum-intensity projection images of PI fluorescence. Scalebars = 16 µm. (**d**) For samples as in (**c**), area (**d**) and perimeter (**e**) of the cells were measured using Fiji. For (**d**) and (**e**), 4–5 cells per image were measured from 25 to 30 images per treatment from three independent experiments. Boxes indicate the median and interquartile range and whiskers indicate minimum and maximum data distribution. Different letters denote statistically significant difference based on ordinary one-way ANOVA and Tukey’s multiple comparisons test (*p* < 0.05).

Isolation of apoplast fluid also will enable studies of its composition, including in response to biotic and abiotic stresses. For such a purpose, an ideal apoplast extraction solution will solvate a maximum diversity of molecules in the apoplast while simultaneously not damaging the plasma membrane of the plant cells and thus contaminating apoplast preparations with cellular contents. Previous studies used water as the apoplast extraction solution^11,12^, which was not expected to cause any significant loss of cellular integrity. Previous work from our lab determined that a 20% methanol (v/v) solution could be used for extraction of the apoplast contents from maize seedling leaves^14^. Therefore, we assessed the effect of including methanol (10, 20 and 50%) in the apoplast extraction solution on yield and cellular integrity during apoplast isolation from Arabidopsis leaves (Fig. 5). The yield of apoplast fluid with 10% or 20% methanol did not differ significantly from that with water, while that with 50% methanol increased significantly to more than 200% (Fig. 5a). We suspected that this large increase in yield with 50% methanol resulted from a loss of cellular integrity resulting in the isolation of both apoplast and cellular contents. Consistent with this supposition, electrolyte leakage did not differ following infiltration with water or 10 or 20% methanol, but increased significantly following infiltration with 50% methanol (Fig. 5b). In addition to indirect assessment by electrolyte leakage, the effects of infiltration with the different percentages of methanol were assessed directly by including propidium iodide (PI) in the infiltrate and analyzing palisade mesophyll cell morphology by confocal microscopy. Overall, we observed that mesophyll cells of leaves infiltrated with water or 10% or 20% methanol did not differ, while those from leaves infiltrated with 50% methanol were deformed (Fig. 5c). Measurements of cell area (Fig. 5d) and perimeter (Fig. 5e) confirmed that infiltration of leaves with 50% methanol reduced the size of mesophyll cells relative to those infiltrated with water or 10 or 20% methanol. Thus, the increases in fluid isolated during the apoplast preparation and in the amount of electrolyte leakage following infiltration with 50% methanol are consistent with the effects of 50% methanol on mesophyll cell morphology. Overall, these results lead us to conclude that, in cases where the goal is to isolate apoplast contents that are poorly soluble in water, such as phenolics, a maximum of 20% methanol should be included within the apoplast extraction solution.

## Discussion

Here we establish an apoplast extraction method optimized for leaves of ~ 5 week-old, vegetatively growing Arabidopsis plants, which advances previously described apoplast extraction methods by adding conditions specific for a leaf type commonly used in the study of Arabidopsis-pathogen interactions, controlling for plant cell integrity following the fluid isolation, and providing an apparently quantitative yield of the apoplast contents^13,17^. Our apoplast isolation method does not cause measurable damage to the cellular integrity of the leaves. Moreover, because our method extracts nearly all of the apoplast contents, we were able to calculate apoplast hydration, which is expressed as the percent to which the apoplast is filled with liquid prior to carrying out the isolation procedure. For plant pathology studies, quantitative measurement of apoplast hydration is an advancement relative to scoring based upon macroscopic observation of WS. Indeed, apoplast hydration during *Pto* DC3000 infection was significant, relative to the non-WS ∆*avre1 ∆hopm1* mutant, at times when WS was not apparent. Notably, apoplast hydration caused by the T3Es AvrE1 and HopM1 correlated with their promotion of *Pto* DC3000 growth and preceded necrotic lesions or evidence for loss of cell integrity through measurements of ion leakage. Thus, *Pto* DC3000 is driving water accumulation in the apoplast during the biotrophic phase of infection. The method we describe will be useful for determining the generality of this observation for other plant–microbe interactions. Beyond analyses related to plant pathology, we anticipate that this method will be useful for studying the water content of leaves of genetically distinct Arabidopsis plants subjected to various stresses and environmental conditions.

In addition to measurements of apoplast hydration, we demonstrate additional applications for our apoplast isolation procedure. The isolation of bacterial cells along with the apoplast fluid provides opportunities for a variety of analysis of the bacteria immediately following their residence *in planta*, such as analysis of the bacterial transcriptome or proteome. This might also be useful for examination of endophytic bacteria populations, assuming the expected low yields can be overcome by sensitive methods of analysis. For approaches of this type, our data do provide a cautionary note. Isolation of the bacteria is likely less than quantitative. And, given the difference we observed in isolation efficiency between bacterial strains and time points, discrete subpopulations of cells may be isolated with differing efficiencies.

Based on our previous work in maize^14,18^, we suggest that the procedure developed here will provide a similarly robust tool for analytical measurements of the apoplast contents of Arabidopsis leaves. The near-complete isolation of apoplast fluid allows for a full accounting of apoplast contents and adds rigor to assessments of changes in those contents. Furthermore, because the method permits quantitation of the amount of apoplast fluid prior to the isolation procedure, the *in planta* concentration of contents of the apoplast can be accurately determined. Such advantages will be useful for a variety of studies, including analysis of the apoplastic response to various biotic or abiotic stresses and between different varieties of plants and mutant plants. To further facilitate such analyses, the use of up to 20% methanol as the wash solution may facilitate the isolation of poorly water-soluble constituents from the apoplast. Thus, we anticipate that this method will facilitate a variety of studies extending beyond analysis of WS during pathogenic infections.

## Materials and methods

### Plant materials and growth conditions

Col-0 Arabidopsis seeds were germinated and grown at ~ 100% relative humidity (RH), 22 °C in an 8-h light/16-h dark cycle photoperiod at 85 μmol m^−2^ s^−1^ for 12–15 days. Then, the seedlings were transplanted into individual pots and grown at RH 65%, in an 8-h light at 225 μmol m^−2^ s^−1^, 22 °C/16-h dark, 16 °C cycle for 3–4 weeks. Bacteria-infected plants were kept at RH ~ 95% (clear dome on) in the same light and temperature cycle.

### Bacteria and growth conditions

*Pto* DC3000 wildtype and *hrcC*^*−*^ mutant strains were grown in King’s B Medium (KB) plates with Rifampicin (75 µg/mL) selection. The *∆avre1 ∆hopm1* strain was grown in KB plates with Rifampicin (75 µg/mL) and Kanamycin (50 µg/mL) selections. All plates were incubated at 25 °C.

### Apoplast extraction

Three fully expanded leaves and ~ 2 mm of petiole (typically leaves 8, 9, and 10) per plant from 3 plants were excised and the IW was recorded immediately. Apoplast wash fluid or water was vacuum-infiltrated in a 30 cc syringe containing ~ 20 mL of apoplast extraction solution as described in main text. The fully saturated leaves were wiped dry thoroughly by blotting with tissue paper wipes and AIW was recorded immediately. The leaves were split into three 3-leaf sets, stacked together, gently rolled, and inserted into 2 mL microcentrifuge tubes containing nine 3.2 mm sterile metal beads. The tubes were left on ice until all the samples were processed and then centrifuged at at 4 °C for 10 min at 7500 rcf (or the designated rcfs in Fig. 2). Then the leaves were gently removed from the tubes using forceps and ASW was recorded immediately.

### Bacterial growth assays

Syringe-infiltration with *Pto* DC3000 strains (at OD_600_ = 0.2), collection of leaf discs and quantification of bacterial growth using serial dilution plating were performed as previously described^19^, except that two leaf punches were added to 2 mL tube with 200 μl sterile water and a 5 mm glass bead and the tissue was ground in a Tissue Lyser (Qiagen) for 1 min at 30 Hz, before serial dilution plating.

### Bacterial counts after apoplast extraction

The efficiency of bacterial extraction along with the apoplast fluid was measured at 7 hai and 15 hai with the indicated strains at OD_600_ = 0.2. After centrifugation to isolate apoplast fluid and bacteria, leaves were weighed, a 0.79 cm^2^ leaf punch was collected from each leaf, and these punches were weighed. The leaf punches were added to a 2 mL tube with 200 μl sterile water and a 5 mm glass bead and the tissue was ground in a Tissue Lyser (Qiagen) for 1 min at 30 Hz. The viable bacteria remaining in the leaf punches was quantified by serial dilution plating and the number of bacteria per leaf mass was calculated. To determine the number of viable bacteria in the isolated apoplast, we first determined the amount of isolated apoplast fluid by adding 500 μl of sterile Milli-Q water to the tubes containing the extracted apoplast, weighing the tubes, and comparing to the weight of the tubes measured prior to the experiment. Then, following vortexing for 10 s, the number of bacteria in the tube was quantified by serial dilution plating and the number of bacteria per leaf mass was calculated. For the total bacterial counts (Fig. 3b), the quantities of bacteria remaining in the leaves and the extracted bacteria were combined. The percent of bacteria extracted and remaining in leaf (Fig. 3c) were calculated as:

$$Percent\;bacteria\;in\;apoplast = \frac{extracted\;bacteria}{{extracted\;bacteria + bacteria\;remaining\;in\;leaf}} \times 100\%$$

$$Percent\;bacteria\;remained\;in\;the\;leaf = \frac{bacteria\;remaining\;in\;leaf}{{extracted\;bacteria + bacteria\;remaining\;in\;leaf}} \times 100\%$$

### Bacterial pellet weights

The weights of the bacterial pellets were measured at 15 hai with the indicated bacterial strains. After centrifugation to isolate apoplast fluid and bacteria into pre-weighed tubes, the metal beads were placed into a sterile 0.5 mL tube with a hole in the bottom and centrifuged again back into the original tube to capture any liquid adhered to the beads. The tubes were then weighed with all apoplast contents and again after the apoplast fluid was removed. The percent of apoplast contents composed of bacteria was calculated as,

$$Percent\;bacteria\;compose\;of\;isolated\;apoplast = \frac{weight\;of\;extracted\;bacterial\;pellet}{{weight\;of\;total\;extracted\;apoplast}} \times 100\%$$

For each experiment, calculated percent bacterial pellet weights were corrected with the averages of water infiltrated samples.

### Confocal imaging

Visualization of propidium iodide staining was carried out using a custom Nikon TiE inverted microscope (Melville, NY) equipped with PerkinElmer UltraVIEW Vox CSUX1 spinning disc unit (Waltham, MA) and using a 40X-silicon oil objective. Images were captured with a DS-QI1 Nikon cooled digital camera using Volocity software (Ontario, Canada).

For propidium iodide staining, fully expanded leaves of 5.5 week-old plants were vacuum-infiltrated with sterile Milli-Q H_2_O, 10% methanol, 20% methanol, or 50% methanol as indicated above for apoplast wash fluid. The leaves were gently blotted dry with tissue paper wipes and 0.2 cm^2^ leaf punches were collected across the midvein by 10 min after infiltration. Leaf punches were floated in 0.5 × MS media with the adaxial surface facing the liquid until all the samples were processed. The MS solution was replaced with 10 μg/mL PI stain (Invitrogen product no. P3566 diluted in sterile Milli-Q H_2_O) and stained at room temperature in the dark for 30 min with three leaf punches per well. Leaf punches were rinsed and floated in 0.5 × MS until imaged using SDCM. Propidium iodide was excited with a Spectra-Physics 561-nm diode laser. Camera exposure was set to 100 ms. Z series of 50 images were taken with a total Z depth of 20 μm. All the Z series images were collapsed to have maximum intensity projections of the images and analyzed using Fiji. The mesophyll cells were traced using a free hand tool and the cell area and perimeter of the cell were measured. Four to five cells per image and 25–30 images per treatment were used in the quantification.

### Conductivity

To assess the initial conductivity of samples, leaves were submerged in 25 mL of Milli-Q H_2_O for one hour with occasional gentle agitation. The conductivity of the water was measured with a Cond 330i meter (WTW) and TetraCon 325 probe (WTW). Blank water conductivity values were subtracted, and then the resulting values were normalized to the initial leaf weight before syringe-infiltration. Averages were obtained from three independent measurements. The maximum conductivities were assessed by measuring after the samples were boiled in a water bath for 20 min and let sit at room temperature for 16 h. The percent electrolyte leakage for individual samples was calculated as:

$$Percent\;Electrolyte\;leakage = \frac{Initial\;conductivity}{{Maximum\;conductivity}} \times 100\%$$

### Statistical analysis

Unless stated otherwise, each experiment was done at least three independent times with similar results. Statistical significances were based on unpaired Two-tailed Welch’s t-test or ordinary one-way ANOVA as stated in the figure legends. Statistical significances were determined with GraphPad Prism 9 software (La Jolla, CA). Grubs test with the Alpha = 0.05 (standard) was performed on the data sets to calculate outliers using GraphPad QuickCalcs outlier calculator (https://www.graphpad.com/quickcalcs/Grubbs1.cfm).

## Supplementary Information

Supplementary Information.

## Data Availability

Beyond the raw data presented in Supplemental Table 1, all the other datasets used and/or analysed during the current study available from the corresponding author on reasonable request.
