# Supplementary material for: A method for quantitation of apoplast hydration in Arabidopsis leaves reveals water-soaking activity of effectors of Pseudomonas syringae during biotrophy
Source: Sci Rep. 2022 Nov 1;12:18363. doi: 10.1038/s41598-022-22472-x (PMC9626588; doi:10.1038/s41598-022-22472-x)
Supplement: Supplementary file 1 — Supplementary Information. [file 41598_2022_22472_MOESM1_ESM.pdf]

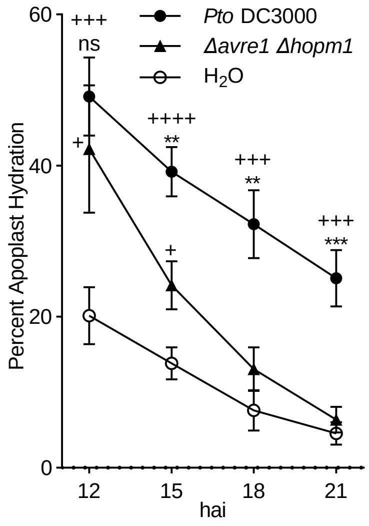

Supplemental figure 1: AvrE1- and HopM1-dependent apoplast hydration.

Leaves of 5 week-old Col-0 plants were syringe-infiltrated with *Pto* DC3000 or *Pto* Δ*avre1* Δ*hopm1* (at OD_{600} = 0.2) or with sterile milliQ water. Apoplast hydration was measured at the indicated time points. For each experiment, n = 3 samples/treatment/time point, with each n consisting of 9 leaves taken from 3 different plants. The experiment was performed 5 times and combined data (n = 15) is shown. Values are means ± SEM and statistically significant differences are based on Welch's t-test. For comparisons of bacterial strains and H_2O; ++++ p<0.0001; +++ p<0.0009; ++ p<0.005; p<0.05. For comparisons between *Pto* DC3000 and Δ*avre1* Δ*hopm1*; **** p<0.0001; *** p<0.0009; ** p<0.005; * p<0.05.

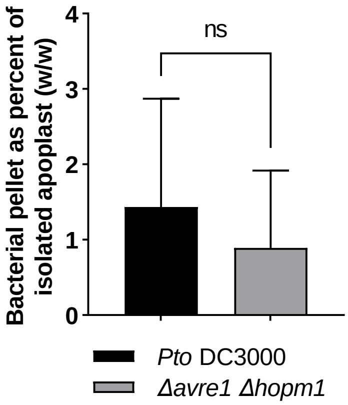

Supplemental Figure 2: Percent of extracted apoplast contents composed of bacteria.

Leaves of 5 week-old Col-0 plants were syringe-infiltrated with *Pto* DC3000 or *Pto* Δ*avre1* Δ*hopm1* (at OD_{600} = 0.2) or with sterile milli-Q water. Apoplast extraction was performed at 15 hai. The percent of total isolated apoplast contents composed of bacteria was determined after correction for evaporation during sample processing of water-infiltrated samples. For each experiment, n = 2 samples/treatment, with each consisting of 9 leaves taken from 3 different plants. The experiment was performed 3 times and combined data (n = 6) is shown. Values are means ± SEM. Lack of statistically significant difference is based on Welch's t-test, p = 0.8553.

Time point (hai)	Sample Type/Number	Initial Weight (g)	After Infiltration Weight (g)	After Spin Weight (g)	Percent Apoplast Hydration
12	DC3000-1	0.3912	0.4695	0.333	42.6
	DC3000-2	0.3991	0.465	0.3251	52.9
	DC3000-3	0.4681	0.5438	0.3913	50.4
	Δavre1 Δhopm1-1	0.3936	0.5157	0.3777	11.5
	Δavre1 Δhopm1-2	0.3386	0.4299	0.3068	25.8
	Δavre1 Δhopm1-3	0.3388	0.4461	0.3182	16.1
	H_2O-1	0.3916	0.5243	0.381	7.4
	H_2O-2	0.421	0.5263	0.3841	25.9
	H_2O-3	0.3626	0.4559	0.3262	28.1
15	DC3000-1	0.3678	0.4602	0.3366	25.2
	DC3000-2	0.3091	0.3647	0.2627	45.5
	DC3000-3	0.3355	0.3746	0.2613	65.5
	Δavre1 Δhopm1-1	0.3452	0.4374	0.3088	28.3
	Δavre1 Δhopm1-2	0.3265	0.4116	0.292	28.8
	Δavre1 Δhopm1-3	0.309	0.3918	0.2785	26.9
	H_2O-1	0.3577	0.4589	0.3361	17.6
	H_2O-2	0.369	0.4825	0.344	18.1
	H_2O-3	0.397	0.5253	0.377	13.5
18	DC3000-1	0.3693	0.4726	0.3357	24.5
	DC3000-2	0.3419	0.4499	0.3083	23.7
	DC3000-3	0.3017	0.3894	0.2714	25.7
	Δavre1 Δhopm1-1	0.3788	0.4938	0.3531	18.3
	Δavre1 Δhopm1-2	0.3542	0.4874	0.3353	12.4
	Δavre1 Δhopm1-3	0.3718	0.5039	0.3583	9.3
	H_2O-1	0.3742	0.5008	0.3646	7.0
	H_2O-2	0.3183	0.4149	0.3072	10.3
	H_2O-3	0.3251	0.4312	0.3189	5.5
21	DC3000-1	0.301	0.4147	0.2815	14.6
	DC3000-2	0.3596	0.4927	0.2991	31.3
	DC3000-3	0.3468	0.4833	0.3147	19.0
	Δavre1 Δhopm1-1	0.3632	0.4939	0.3486	10.0
	Δavre1 Δhopm1-2	0.3446	0.4927	0.3376	4.5
	Δavre1 Δhopm1-3	0.3039	0.4485	0.3015	1.6
	H_2O-1	0.3245	0.4313	0.3143	8.7
	H_2O-2	0.44	0.6042	0.4399	0.1
	H_2O-3	0.3669	0.5153	0.3634	2.3

Supplemental Table 1: Raw data from an apoplast hydration assay.

Shown is the raw data from one of the five experimental repeats comprising the results presented in Figure 3c and Supplemental Figure 1. Leaves of 5 week-old Col-0 plants were

syringe-infiltrated with either *Pto* DC3000 or *Pto* Δ*avre1* Δ*hopm1* (at OD_{600} = 0.2) or with sterile milliQ water. The three technical replicates (indicated as -1, -2 and -3) for individual treatments/time points each consisted of 9 leaves from 3 separate plants. The initial, after infiltration, and after spin weights were recorded and used to calculate the percent apoplast hydration. hai = hours after infection.
